# A longitudinal study of the mental health of autistic children and adolescents and their parents during COVID-19: Part 1, quantitative findings

**DOI:** 10.1177/13623613221082715

**Published:** 2022-06-06

**Authors:** Umar Toseeb, Kathryn Asbury

**Affiliations:** University of York, UK

**Keywords:** autism, COVID-19, longitudinal, mental health, mixed-methods, qualitative, quantitative, special educational needs

## Abstract

Autistic children and adolescents and their parents are likely to have been disproportionally affected during the COVID-19 pandemic. There has been little focus on how the mental health of these vulnerable families developed during the pandemic and how it compared to those with other special educational needs and disabilities. Questionnaires were completed by a maximum of 527 parents/carers about their own and their child’s mental health at one or more time points between 23 March 2020 (at the onset of the first lockdown) and 10 October 2020 (when schools fully reopened for face-to-face teaching). Multi-level regression models were fitted to the data. Autistic young people had more depression and anxiety symptoms compared to young people with other special educational needs and disabilities throughout the study period. As lockdown progressed and schools subsequently reopened for face-to-face teaching, anxiety levels decreased for young people with special educational needs and disabilities but not for autistic young people, whose anxiety levels remained stable throughout. Depression symptoms, however, remained stable for both groups during this period as did parents’/carers’ psychological distress and well-being. These findings shed new light on the likely disproportionate effect of the COVID-19 pandemic on anxiety levels in autistic young people.

**Lay abstract:**

Autistic children and adolescents, and their parents/carers, tend to experience more symptoms of anxiety and depression compared to those with other special educational needs and disabilities. The rapid change in society as a result of the COVID-19 pandemic is likely to have disproportionately affected autistic young people and their parents/carers. We investigated how the mental health of autistic young people, and their parents/carers, developed during the first lockdown in the United Kingdom and how it changed once schools fully reopened for face-to-face teaching approximately 6 months later. Parents/carers completed online standardised questionnaires about their own and their child’s mental health at four time points between March 2020 and October 2020. We found that, throughout this period, autistic young people experienced more symptoms of anxiety and depression compared to those with other special educational needs and disabilities. Anxiety levels decreased as lockdown progressed and schools reopened for face-to-face teaching but only for those with other special educational needs and disabilities. For autistic young people, both anxiety and depression symptoms remained high throughout. There were no differences in the mental health of parents/carers of autistic children compared to those with other special educational needs and disabilities. These findings suggest that the mental health of autistic children and adolescents is likely to have been disproportionately affected during and after the first lockdown in the United Kingdom. In the second part of this article (Asbury & Toseeb, 2022), we attempt to explain these trends using qualitative data provided by parents during the same period.

Autistic children and adolescents tend to have co-occurring psychiatric conditions. Autism is characterised by social and communication difficulties and restrictive and repetitive behaviours, interests and activities (American Psychiatric Association (APA), 2013). Many autistic young people rely on carefully established support networks and routines, which were abruptly disrupted by the onset of the COVID-19 pandemic. The first United Kingdom (UK) COVID-19 lockdown began on 23 March 2020 – school buildings were closed, teaching was rapidly moved online for most pupils and everything other than essential services was forced to shut. This seismic shift in everyday life is likely to have affected the mental health of autistic young people and their families. In the current study, online questionnaires were used to investigate how the mental health of autistic children and adolescents, and their parents/carers, changed during and after the first COVID-19 lockdown in the UK, and how it compared to those with other special educational needs and disabilities (SENDs).

## Mental health of autistic young people

Mental health difficulties are more common in autistic young people compared to the general population. The number of autistic young people with a diagnosable anxiety disorder is approximately double when compared to the general population (Beesdo et al., 2009; van Steensel et al., 2011). For depressive disorders, autistic young people have a fourfold increase in lifetime risk (Hudson et al., 2019). These group-level differences in diagnostic rates also exist at a symptom level. Similarly, autistic young people have higher rates of mental health difficulties compared to those with other types of SENDs such as conduct disorder (Green et al., 2000) and attention-deficit hyperactivity disorder (van Steensel et al., 2013). This might be because autistic young people experience high levels of risk factors and fewer protective factors, and are therefore more likely to experience mental health difficulties, than other young people. They are more likely to be bullied by siblings (Toseeb et al., 2018) and peers (Toseeb, McChesney, et al., 2020), they have more communication difficulties (APA, 2013) and they have poorer quality friendships (Sedgewick et al., 2019), all of which are associated with heightened mental health difficulties. More specifically, the intolerance of uncertainty, finding it difficult to cope with the unexpected, is associated with anxiety in autistic people (Jenkinson et al., 2020). This may have been a particularly salient risk factor during the COVID-19 pandemic.

The experiences of depression and anxiety symptoms may also be qualitatively different in autistic young people compared to the general population. Autistic young people experience sensory symptoms in differing frequency and intensity compared to their neurotypical peers – they may be under- or over-sensitive to sensory symptoms (Ben-Sasson et al., 2009). They may also have different coping strategies such that they more frequently adopt poorer emotional regulation strategies (Mazefsky et al., 2013). Therefore, the mental health of autistic children and adolescents during the COVID-19 pandemic may have been affected in a qualitatively and quantitatively different way compared to neurotypical children or those with other SENDs.

## Mental health of autistic young people during COVID-19

There is some limited evidence on how the COVID-19 pandemic has affected the mental health of autistic young people. Most of this evidence comes from studies outside the UK. Two such studies, one from Ireland (O’Sullivan et al., 2021) and the other from Portugal (Amorim et al., 2020), highlighted disruption to routines as a key driver of increase in anxiety for autistic young people compared to those who are not autistic. Studies that relied on parents to retrospectively compare their child’s mental health to pre-pandemic levels generally show a worsening of mental health (e.g. Colizzi et al., 2020; Masi et al., 2021; Nuñez et al., 2021). However, those that directly compared young people’s mental health during the COVID-19 pandemic to measures taken before the pandemic show mixed results. Findings from studies in Italy (Siracusano et al., 2021) and Spain (Lugo-Marín et al., 2021) suggest that there was no significant deterioration of mental health in autistic young people compared to pre-pandemic levels. Research from the United States of America (Vasa et al., 2021) and Turkey (Mutluer et al., 2020), however, found a significant increase in mental health difficulties compared to pre-pandemic levels. These differences in findings may be a result of methodological differences such as sample size, measures, study design and so on. They may also be reflective of each country’s handling of the pandemic. Young people’s mental health is influenced by a complex interplay of individual, social and environmental factors (Bronfenbrenner, 1979). Therefore, wider government responses to the pandemic and the implemented policies may have a direct or indirect effect on young people’s mental health. The importance of these country-level factors may have been more pronounced for families with autistic children, who had a number of unmet needs during the first COVID-19 lockdown in the UK (Toseeb, Asbury et al., 2020).

To the best of the authors’ knowledge, the first study on the mental health of autistic young people in the UK was published by Asbury, Fox, Deniz, Code and Toseeb (2021). They asked parents of children with SENDs (over 80% autistic) about the effect of the COVID-19 pandemic on their child’s mental health. Parents described their child was experiencing loss and worry and reported deterioration in mood and behaviour. A similar set of findings were presented in a separate study by Banerjee, Khan and Kesavan (2021). Both of these studies provide preliminary evidence of the negative effects of COVID-19 pandemic on autistic young people but without a formal comparison to neurotypical samples or those with other types of SENDs it is not possible to determine whether these difficulties were more pronounced for autistic young people. In a separate study, it was found that autistic young people had poorer mental health compared to neurotypical young people but not necessarily poorer than young people with other types of SENDs (Nonweiler et al., 2020). Therefore, there is a dearth of research comparing the mental health of autistic young people with young people with other types of SENDs during the COVID-19 pandemic.

## Mental health of parents/carers of autistic young people

Parents/carers of autistic children and adolescents are more likely to experience mental health difficulties than other parents/carers. They fare worse on various measures of mental health compared to parents of neurotypical children (e.g. Hoffman et al., 2009) and those of children with other types of SENDs (e.g. Pisula, 2007). Given the high level of additional needs that some autistic young people have, parents/carers may experience additional stress, exhaustion or burnout, contributing to an increased risk of mental health difficulties (Quintero & McIntyre, 2010). Even prior to the COVID-19 pandemic, the additional care that an autistic young person requires could lead to additional stress in parents/carers (DePape & Lindsay, 2014). Similarly, parents/carers are expected to manage the additional behavioural difficulties that are common in autistic young people, which may lead to additional stress and, in turn, mental health difficulties (Davis & Carter, 2008). These additional stressors are likely to have been amplified during the COVID-19 pandemic when support networks were broken, routines were abruptly changed, parents/carers were expected to meet all of the additional needs of their child or young person and so on, without the usual support.

## Mental health of parents/carers of autistic young people during COVID-19

There are a handful of studies focussing on the impact of COVID-19 on the mental health of parents of autistic young people. There is some evidence that the mental health of parents of autistic children declined after the onset of the COVID-19 pandemic (Althiabi, 2021). This decline in mental health appears to have been greater for parents of autistic young people compared to parents of neurotypical young people (Pecor et al., 2021). Even during the pandemic, parents of autistic young people had poorer mental health compared to parents of neurotypical young people (Wang et al., 2021). The pandemic led to a number of changes in the level of support available to parents of autistic children and adolescents. It is not surprising, therefore, that parents’ mental health during the pandemic was correlated with the frequency and usefulness of support (Alhuzimi, 2021). Similarly, parents’ own resilience and coping strategies affected their mental health during the COVID-19 pandemic (Wang et al., 2021). The current literature, therefore, suggests that the COVID-19 pandemic has had a disproportionate effect on the mental health of parents/carers of autistic young people. Given that parents’ and child’s mental health are highly correlated, it is likely that parents’ mental health influences child’s mental health and vice versa.

## The current study

The evidence regarding the effects of the COVID-19 pandemic on the mental health of autistic young people and their parents/carers is beginning to emerge but as expected, it is very limited. To the best of the authors’ knowledge, all of the studies to date that have directly compared autistic young people with neurotypical children or those with other SENDs have been conducted outside the UK. There are some studies from the UK that have combined samples of children and autistic young people with other types of SENDs but this is problematic as autistic children and adolescents face unique stressors and challenges and therefore warrant separate investigations. There is one notable exception (Nonweiler et al., 2020), but this study is also limited because it was cross-sectional and therefore does not allow for an investigation of how mental health developed during the COVID-19 pandemic. As autistic young people began to adjust to a new routine and the support mechanisms started to be reinstated, mental health of parents and children may have improved. In addition, Nonweiler et al. (2020) used the Strengths and Difficulties Questionnaire (Goodman, 1997), which is a non-specific measure of mental health. Given that autistic young people may experience mental health difficulties differently, this measure may not have captured the diversity and complexity of mental health experiences of autistic children and young people during the COVID-19 pandemic. To address these gaps in the literature, the current study aimed to:

1. Map the longitudinal trajectories of symptoms of depression and anxiety in autistic children and adolescents during and after the first COVID-19 lockdown in the UK (Aim 1). The research questions were as follows:
  Research Question 1a: To what extent did symptoms of depression and anxiety change for autistic children and adolescents from the beginning of the first COVID-19 lockdown until after school return, 6 months later?
  Research Question 1b: Were the symptoms of depression and anxiety different for autistic children and adolescents compared to those with other types of SENDs?
  Research Question 1c: Which factors predicted symptoms of depression and anxiety for autistic children and adolescents?
2. Map the longitudinal trajectories of psychological well-being and distress in parents of autistic children and adolescents during and after the first COVID-19 lockdown in the UK (Aim 2). The research questions were as follows:
  Research Question 2a: To what extent did parents’/carers’ psychological well-being and distress change from the beginning of the first COVID-19 lockdown until after school return, 6 months later?
  Research Question 2b: Was psychological well-being and distress different for parents of autistic children and adolescents compared to children with other types of SENDs?
  Research Question 2c: Which factors predicted parents’ psychological well-being and distress?

## Method

### Ethics

The study was approved by the Education Ethics Committee at the University of York (Reference 20/05). Parents/carers of children and adolescents provided informed consent electronically.

### Participant recruitment and study design

A maximum of 527 parents/carers of autistic young people (75%) and other SENDs completed online questionnaires between 22 March 2020 and 10 October 2020. Initial recruitment yielded 310 parents/carers and a further 217 were recruited at follow-up stages. Those with more than one autistic child or other SENDs were asked to focus on just one child in their responses. Email invites were sent via a number of recruitment streams. These included existing research networks (e.g. Autistica and the National Autistic Society), alternative provision schools (e.g. special schools, pupil referral units, etc.), online social media platforms (e.g. Twitter or Facebook groups) and a paid research site (i.e. Prolific).

The study design was intended to be a longitudinal cohort study but it turned out to be quite complex. During 2020, data were collected at four time points: Time 1 (23 March 2020–22 April 2020), Time 2 (23 April 2020–22 May 2020), Time 3 (23 May 2020–22 June 2020) and Time 4 (29 September 2020–10 October 2020). The first UK lockdown started on 23 March 2020 when face-to-face teaching was ceased for all but the most vulnerable children (including some children with SENDs), non-essential shops were closed, and people were advised to work from home, if possible. A phased reopening of schools began on 01 June 2020 and non-essential shops were allowed to reopen on 15 June 2020. In September 2020, face-to-face teaching resumed for all children. Parents took part at one or more of four time points. Those who took part at one time point were invited to take part in all future time points. Given the level of sample attrition, new participants were recruited at each time point to boost the sample size and maximise power. Full details of missing data and sample attrition are provided in the supplementary materials.

### Measures

#### Demographic information

##### Relationship to the young person

Parents/carers were asked ‘What is your relationship to your child?’ and responded by selecting one of three options (0 = *Mother*, 1 = *Father*, 2 = *Other*).

##### Region of the UK

Parents/carers were asked ‘Which part of the UK do you live in?’ and responded by selecting one of four options (0 = *England*, 1 = *Scotland*, 2 = *Northern Ireland*, 3 = *Wales*).

##### Household income

Parents/carers were asked ‘What is your household income before tax?’ and responded by selecting one option from a list (£0–£9999, £10,000–£19,999, £20,000–£29,999, £30,000–£39,999, £40,000–£49,999, £50,000–£59,999, £60,000–£69,999, £70,000–£79,999, £80,000 or more). Given that the UK median household income is approximately £40,000 (pre-tax), responses were recoded into a binary variable (0 = *below median income*, 1 = *above median income*).

##### Age

Parents/carers were asked ‘How old is your child (years)?’ and selected an option of between 5 and 18 years from a drop-down list.

##### Sex

Parents/carers were asked ‘Is your child a . . . ’ and selected one of the following options: ‘boy’, ‘girl’ or ‘other’. Responses were coded into a binary variable (0 = *girl*, 1 = *boy*).

##### Ethnicity

Parents/carers were asked about their child’s ethnicity ‘What is your child’s ethnicity?’ and responded by selecting one of the following: Asian (Bangladeshi, Chinese, Indian, Pakistani, Asian Other), Black (Black African, Black Caribbean, Black Other), Mixed (Mixed White/Asian, Mixed White/Black African, Mixed White/Black Caribbean, Mixed Other), White British, White Non-British (White Irish, White Gypsy/Traveller, White European, White Other) or Other (Arab, Any Other). Due to the small numbers of participants in all categories except White British, these were recoded to create a binary scale (0 = *White British*, 1 = *ethnic minority*).

##### Type of school

Parents/carers were asked ‘What type of school does your child attend?’ and responded by selecting one of four options (0 = *mainstream school*, 1 = *special school*, 2 = *pupil referral unit*, 3 = *other*). These responses were recoded to create a binary variable (0 = *mainstream school*, 1 = *alternative provision*).

##### Education, health and care plan

Parents/carers were asked ‘Does your child have an education, health and care plan (EHCP)?’ and responded by selecting one of two options (0 = *no*, 1 = *yes*). An EHCP is a legal document that sets out the educational, health and social care needs of a young person with SENDs. In general, all children with an EHCP have additional learning needs but only those whose needs cannot be met by existing support have an EHCP.

##### Type of SENDs

Given that SENDs co-occur, parents were given a list of common SENDs and asked to select all that applied. The question was ‘What types of special educational needs or disabilities does your child have? Select all that apply’.

##### Young person verbal ability

Parents/carers were asked ‘which of the following statements best describes your child?’ and the response options were ‘can speak several words or speaks in sentences’ or ‘uses few or no words’. Those who responded affirmatively to the latter were excluded from this analysis as the quantitative mental health measures were not administered to these families.

#### Young person mental health

##### Anxiety symptoms

Parents reported on their child’s anxiety using the anxiety scale for children with autism spectrum disorder (Rodgers et al., 2016). The scale is an adapted version of the Revised Child Depression and Anxiety Scale (Chorpita et al., 2000). It considers the phenomenology of anxiety of autistic individuals. The scale consists of 24 items with a four-point response scale (0 = *never*, 1 = *sometimes*, 2 = *often*, 3 = *always*). The scale consists of five items on performance anxiety (e.g. my child worries when he or she thinks he or she has done poorly at something in case people judge him or her negatively), six items on anxious arousal (e.g. my child suddenly feels so anxious he or she feels as if he or she cannot breathe when there is no reason for this), five items on separation anxiety (e.g. my child worries that something awful will happen to someone in the family) and eight items on uncertainty anxiety (e.g. my child always wants to know what will happen next). An overall anxiety score was generated, which combines responses to all 24 items into a single score with higher scores indicating higher levels of anxiety.

##### Depression symptoms

Parents reported on their child’s depression symptoms using the low mood subscale of the Revised Child Depression and Anxiety Scale (Chorpita et al., 2000). The scale consists of ten items with a four-point response scale (0 = *never*, 1 = *sometimes*, 2 = *often*, 3 = *always*). Sample items were ‘my child feels sad or empty’ and ‘my child has no energy for things’. Responses were summed to create a single score for each child with higher scores indicating more depression symptoms.

#### Parent/carer mental health

##### Psychological distress

Parents/carers reported on their own symptoms of negative mental health using the self-report Kessler-6 Psychological Distress Scale (Kessler et al., 2003). The scale consists of six statements and respondents are asked to indicate the extent to which they have felt this way in the past 30 days. Example items include ‘nervous’, ‘hopeless’ and ‘worthless’. Responses were provided on a five-point scale (1 = *all of the time*, 2 = *most of the time*, 3 = *some of the time*, 4 = *a little of the time*, 5 = *none of the time*). Parent/carer responses were reverse scored and summed to generate a single score for each respondent. Higher scores indicated higher levels of psychological distress.

##### Well-being

Parents/carers reported on their overall well-being using the short Warwick–Edinburgh Mental Wellbeing Scale (Tennant et al., 2007). The scale consists of seven items asking respondents to reflect on the preceding 2 weeks. Example items include ‘I’ve been feeling optimistic about the future’ and ‘I’ve been thinking clearly’. Responses were given on a five-point scale (1 = *none of the time*, 2 = *rarely*, 3 = *some of the time*, 4 = *often*, 5 = *all of the time*). Responses to the items were scored in line with the scoring guidelines. Higher scores indicated higher levels of well-being.

### Statistical analysis

All statistical models were fitted in STATA/MP 17.0 (StataCorp, 2021). A number of mixed-effect linear regression models were fitted with the maximum likelihood estimator to deal with missing data (Ibrahim & Molenberghs, 2009; Nooraee et al., 2018). The dependent variable was different in each of the models: young person anxiety symptoms (Model 1), young person depression symptoms (Model 2), parent/carer psychological distress (Model 3) and parent/carer well-being (Model 4). The independent variables were the same in all models. In the fixed part of the models, these were group (0 = *SEND*, 1 = *autism*), linear effect of time (T1, T2, T3 and/or T4), interaction term for group and linear effect of time, young person age, young person sex (0 = *girl*, 1 = *boy*), young person ethnicity (0 = *White British*, 1 = *ethnic minority*), family income (0 = *above median*, 1 = *below median*), EHCP (0 = *no*,1 = *yes*) and type of school (0 = *mainstream*, 1 = *alternative provision*). The predictors in the random part of the model were anonymised participant id and the linear effect of time.

For models where the interaction term for linear time and group was significant, a number of sub-models were fitted. First, the models for autism and SENDs were fitted separately to determine whether the change over time was different between groups. Next, the models for each time point were fitted separately to determine whether there were between-group differences within each time point. For these post hoc comparisons, a Bonferroni correction was applied (0.05/9 = 0.006).

### Community involvement

The second article of this study represents voices of parents of autistic children and adolescents (Asbury & Toseeb, 2022). In addition to this, a member of the research team, who was centrally involved in the design of the study and study questions, is a parent of an autistic child.

## Results

### Sample demographics

Parents/carers were asked to self-report demographic information. Detailed sample demographics, divided by time point, are provided in Table 1 but they are described here in brief. Most of the respondents (90%) were mothers of the young person and from England (93%). Approximately, half of the sample (48%) was from households with below median income. In terms of the young people, the mean age was approximately 11 years (range 5–18 years), most were boys (70%) and White British (90%). Over half the sample (57%) was enrolled in mainstream school and the remainder were in a special school, pupil referral unit or were being home-schooled (pre-COVID-19 pandemic). Just under two-thirds of the sample (62%) had an EHCP. Therefore, the sample is representative of the broad UK population with oversampling of those from below median income households, alternative school provision and those with an EHCP.

**Table 1.** Sample demographics.

	Overall	Time 1	Time 2	Time 3	Time 4
Sample size	**517 (100%)**	**310 (100%)**	**229 (100%)**	**189 (100%)**	**191 (100%)**
Autism	389 (75%)	250 (81%)	184 (80%)	130 (69%)	144 (75%)
Special educational needs and disabilities	128 (25%)	60 (19%)	45 (20%)	59 (31%)	47 (25%)
Respondent	**517 (100%)**	**309 (100%)**	**221 (100%)**	**176 (100%)**	**191 (100%)**
Mother	465 (90%)	282 (91%)	208 (94%)	150 (85%)	164 (86%)
Father	36 (7%)	14 (5%)	6 (3%)	22 (13%)	23 (12%)
Other (foster, adoptive, grandparents, etc.)	16 (3%)	13 (4%)	7 (3%)	4 (2%)	4 (2%)
Country within United Kingdom	**507 (100%)**	**309 (100%)**	**228 (100%)**	**183 (100%)**	**188 (100%)**
England	472 (93%)	296 (96%)	218 (95%)	175 (96%)	169 (90%)
Northern Ireland	18 (4%)	7 (2%)	6 (3%)	4 (2%)	9 (5%)
Scotland	11 (2%)	4 (1%)	2 (1%)	2 (1%)	6 (3%)
Wales	6 (1%)	2 (1%)	2 (1%)	2 (1%)	4 (2%)
Household income	**500 (100%)**	**302 (100%)**	**224 (100%)**	**180 (100%)**	**168 (100%)**
Below median income	241 (48%)	159 (53%)	100 (55%)	83 (46%)	71 (38%)
Above median income	259 (52%)	143 (47%)	124 (45%)	97 (54%)	115 (62%)
Young person age (years)	**10.69 (3.34)**	**10.14 (3.27)**	**11.05 (3.25)**	**10.68 (3.48)**	**11.19 (3.33)**
Young person sex	**504 (100%)**	**308 (100%)**	**225 (100%)**	**182 (100%)**	**188 (100%)**
Girl	152 (30%)	94 (31%)	60 (27%)	66 (36%)	55 (29%)
Boy	352 (70%)	214 (69%)	165 (73%)	116 (64%)	133 (71%)
Young person ethnicity	**508 (100%)**	**309 (100%)**	**229 (100%)**	**183 (100%)**	**189 (100%)**
White British	456 (90%)	280 (91%)	206 (90%)	163 (90%)	169 (89%)
Ethnic minority	52 (10%)	29 (9%)	23 (10%)	20 (11%)	20 (11%)
Young person educational placement	**509 (100%)**	**310 (100%)**	**229 (100%)**	**183 (100%)**	**189 (100%)**
Mainstream	291 (57%)	148 (48%)	130 (57%)	103 (56%)	116 (61%)
Alternative provision	218 (53%)	162 (52%)	99 (43%)	80 (44%)	73 (39%)
Young person education health and care plan	**509 (100%)**	**310 (100%)**	**229 (100%)**	**183 (100%)**	**189 (100%)**
No	192 (38%)	102 (33%)	85 (37%)	68 (37%)	75 (40%)
Yes	317 (62%)	208 (67%)	144 (63%)	115 (63%)	114 (60%)

Values represent maximum sample size. Some *N*s differ to other tables due to missing data on specific measures. The values in bold with % are a function of the subheadings below and not of the overall sample size.

Children with a broad range of SENDs were included in the sample. Full details of the range of SENDs at each time point are provided in Table S1 (supplementary materials). Parents/carers of approximately three-quarters of the sample reported that they had an autistic child. Approximately a third reported that their child had social, emotional and mental health difficulties. A fifth reported that their child had attention-deficit hyperactivity disorder. The rest of the SENDs were reported by fewer than a fifth of parents. There was some minor variation in the proportion of SENDs as a function of the total sample at each time point but, on the whole, these proportions were similar across time points. As expected, there was considerable co-occurrence of SENDs across the sample. More than half of parents/carers reported that their child had more than one SEND.

### Descriptive statistics

Descriptive statistics for all of the measures of mental health are shown in Table 2. All of the measures had good or excellent internal consistency (α > 0.8) except young person depression symptoms, which had acceptable internal consistency (α > 0.7).

**Table 2.** Descriptive statistics for quantitative measures of mental health.

	Young person		Parent/carer	
	Anxiety symptoms	Depression symptoms	Psychological distress	Well-being
Range	0–66	0–30	6–30	6–30
Time 1				
Cronbach’s α	0.94	0.73	0.82	0.81
Autism mean (*SD*)	35.16 (15.03)	11.46 (5.74)	14.95 (4.83)	19.09 (4.03)
SEND mean (*SD*)	28.63 (13.34)	9.97 (5.08)	13.65 (4.83)	20.12 (3.80)
Time 2				
Cronbach’s α	0.93	0.73	0.84	0.83
Autism mean (*SD*)	36.40 (13.82)	12.17 (5.09)	14.37 (4.73)	19.18 (3.99)
SEND mean (*SD*)	29.75 (13.98)	11.40 (6.26)	13.81 (5.18)	19.90 (4.11)
Time 3				
Cronbach’s α	0.93	0.73	0.85	0.81
Autism mean (*SD*)	35.38 (13.89)	11.76 (5.16)	13.54 (4.58)	19.32 (3.52)
SEND mean (*SD*)	27.51 (12.87)	9.87 (5.56)	14.60 (5.23)	19.21 (3.77)
Time 4				
Cronbach’s α	0.94	0.79	0.86	0.85
Autism mean (*SD*)	35.24 (14.10)	11.40 (5.53)	14.65 (4.99)	18.97 (4.20)
SEND mean (*SD*)	24.48 (14.80)	9.02 (5.26)	13.72 (4.85)	19.10 (4.15)

SEND = special educational needs and disability, *SD* = standard deviation.

### Young person mental health

Two mixed-effect regression models were fitted to investigate anxiety and depression symptoms for the young person with SENDs during and after the first lockdown (Research Question 1).

#### Anxiety symptoms

As shown in Table 3 (Model 1), there were a number of significant main effects. The interaction term between group and time was also significant, suggesting that anxiety symptoms were dependent on group (autism or SEND). Therefore, the models were fitted separately for each group (see Table S2 in supplementary materials).

**Table 3.** Regression models for quantitative measures of mental health.

	Young person		Parent/carer	
	Model 1: anxiety symptoms	Model 2: depression symptoms	Model 3: psychological distress	Model 4: well-being
Wald χ^2^	χ^2^ (9, 481) = 102.43, *p* < 0.001	χ^2^ (9, 486) = 67.08, *p* < 0.001	χ^2^ (9, 481) = 16.42, *p* = 0.058	χ^2^ (9, 481) = 102.43, *p* = 0.001
Predictor				
Autism	8.67 [5.52, 11.81]***	1.45 [0.30, 2.60]^*^	0.41 [−0.73, 1.55]	−0.86 [−1.76, 0.04]
Linear time	−0.83 [−1.31, −0.35]**	−0.13 [−0.31, 0.05]	−0.04 [−0.24, 0.16]	−0.14 [−0.30, 0.02]
Autism × linear time	0.79 [0.25, 1.33]**	0.06 [−0.15, 0.27]	0.07 [−0.17, 0.31]	0.09 [−0.10, 0.28]
Age	0.61 [0.28, 0.95]***	0.45 [0.31, 0.58]***	0.01 [−0.12, 0.13]	−0.02 [−0.12, 0.08]
Boy	−3.37 [−5.90, −0.84]**	−1.08 [−2.07, −0.09]^*^	0.44 [−0.47, 1.35]	−0.28 [−1.00, 0.44]
Ethnic minority	−3.34 [−7.19, 0.51]	−0.89 [−2.39, 0.61]	0.44 [−0.94, 1.81]	−0.26 [−1.35, 0.83]
Below median income	2.52 [0.22, 4.82]*	0.32 [−0.58, 1.22]	1.48 [0.66, 2.31]***	−1.45 [−2.10, −0.79]***
EHCP	−0.88 [−3.51, 1.75]	0.16 [−0.87, 1.19]	0.34 [−0.61, 1.29]	−0.31 [−1.06, 0.44]
Alternative provision	−2.25 [−4.86, 0.36]	−1.08 [−2.10, −0.06]*	−0.14 [−1.08, 0.79]	0.02 [−0.72, 0.76]

Values are unstandardised beta coefficients [95% confidence intervals]. When there were significant interaction terms, these models were re-run separately by group (autism, SEND) and time (T1, T2, T3, T4). These models are reported in the appendices.

EHCP = education, health and care plan.

* *p* < 0.05, **p < 0.01, ***p < 0.001.

The separate group models revealed a significant main effect of time in the SEND model but not the autism model. This suggests that there was a decrease in anxiety symptoms between T1 and T4 for young people with SENDs but for autistic young people, there was no change in anxiety symptoms between T1 and T4. In addition to this, in the autism model there were significant main effects of age and sex, suggesting that for the autistic group overall anxiety was higher for older children and for girls compared to younger children and boys.

The separate models for each time point revealed a significant main effect of group in all models (see Table S3 in supplementary materials), suggesting the autistic young people had higher levels of anxiety symptoms at all four time points. In addition to this, at Time 1, but not at any other time point, there was a significant main effect of age, suggesting that older children had higher overall levels of anxiety compared to younger children (irrespective of whether they were autistic or had another SEND).

#### Depression symptoms

The general pattern of results for depression symptoms was different to anxiety symptoms (Table 3, Model 2). Autistic young people had more depression symptoms compared to young people with SENDs (main effect of group). This did not change over time (main effect of time not significant) and this lack of change was similar between groups (interaction between group and time not significant). There were also significant main effects of age, sex and school type, suggesting that older children, girls and those in mainstream schools had higher levels of depression symptoms compared to younger children, boys and those in alternative provision, respectively.

### Parent/carer mental health

Two mixed-effect regression models were fitted to investigate psychological distress and well-being of parents/carers during and after the first lockdown (Research Question 2).

#### Psychological distress and well-being

As shown in Table 3, there were no significant main effects of group or time nor were there significant interactions between group and time for either psychological distress (Model 3) or well-being (Model 4). This suggests that parents of young people with SENDs and autistic young people experienced similar levels of psychological distress and well-being at all four time points and there were no significant changes over time. There was also a significant main effect of household income in both models, suggesting that those from below median income households had higher levels of psychological distress and lower levels of well-being compared to those from higher income households.

## Discussion

This was the first study to investigate how the mental health of autistic young people and their parents/carers changed during and after the first COVID-19 lockdown in the UK. As lockdown progressed and schools subsequently reopened for face-to-face teaching, anxiety levels decreased for young people with SENDs but not for autistic young people, whose anxiety levels remained stable throughout. Depression symptoms, however, remained stable for both groups during this period as did parents’/carers’ psychological distress and well-being.

### Mental health of autistic young people

Anxiety is particularly prevalent among autistic young people and this was reflected in the findings of the current study. Throughout the first lockdown, and after, autistic young people had higher levels of anxiety compared to those with SENDs. As the first lockdown progressed, and ended, the difference in anxiety between autistic young people and those with SENDs grew larger. It was driven by a decrease in anxiety symptoms for those with SENDs. Given that autistic young people rely on carefully established routines and support networks, it was not surprising that anxiety in this group was higher at the beginning of lockdown, when these routines and support networks were severely disrupted. But as lockdown progressed it might have been expected that autistic young people would adapt to the ‘new normal’ and that anxiety levels might decrease. In addition, autistic young people who are bullied at school experience higher levels of anxiety (Chou et al., 2020) and so being away from school might have been expected to alleviate some of this. Indeed, previous work in this sample found that for some autistic young people anxiety levels might have been lower than previously because they no longer had the daily torture of school (Asbury et al., 2021). One explanation for why this pattern of decreasing anxiety was not seen in the data is that that source of anxiety changed. For example, ongoing uncertainty about the future and worry about the effect of the virus on the young person and their family replaced previous sources of worry such as the school environment (Asbury et al., 2021). Furthermore, the analysis reported here shows that for autistic young people, attending a mainstream school was associated with higher levels of anxiety (irrespective of time point). It may be that those attending specialist provision were better catered for and this is why their anxiety levels were lower. Indeed, previous work in this sample reports educational activities that were being set for home-school were not always appropriate keeping in mind the young person’s needs and existing support plans (Toseeb, Asbury et al., 2020) and these were affecting the young person’s mental health (Asbury et al., 2021). Furthermore, on return to school, there was no decrease in anxiety for autistic young people. Schools looked very different when students returned in September 2020 – lots of COVID-19 protective measures were in place – all new and therefore potentially a new source of anxiety for autistic young people. Anxiety levels for autistic children and young people remained stable across all time points, in spite of the changing situation with its changing stressors.

As expected, during and after the first COVID-19 lockdown, autistic young people had more depression symptoms compared to young people with SENDs. There was no change in depression symptoms for either group as lockdown progressed and schools subsequently reopened. In other samples, those with SENDs (a distinction between autism and other SENDs was not made) had fewer emotional difficulties as lockdown progressed (Raw et al., 2021). This difference in findings may be due to a difference in the measures used. In the current study, we distinguished between symptoms of depression and anxiety whereas Raw et al. (2021) used a non-specific measure of emotional symptoms. Our findings suggest that anxiety symptoms may be driving the decrease in emotional symptoms reported by Raw et al. (2021). Alternatively, prolonged periods at home may have increased risk factors for poor mental health. Indeed, previous work in this sample found that sibling conflict increased during COVID-19 in SEND families (Toseeb, 2021), which is associated with poorer mental health in autistic young people (Toseeb et al., 2018; Toseeb, McChesney, et al., 2020). Therefore, any improvement in depression symptoms may have been offset by an increase in sibling conflict. Therefore, the change in mental health of children and adolescents during and after the first COVID-19 lockdown was partly dependent on whether they were autistic or had another SEND.

### Mental health of parents of autistic young people

Parents of autistic young people experienced similar levels of psychological distress and well-being throughout and after the first lockdown compared to parents of young people with other SENDs. This is surprising given that pre-pandemic research suggests parents of autistic young people have poorer mental health compared to those of other SENDs (Pisula, 2007). It appears that the additional stressors of the COVID-19 pandemic equally affected parents of autistic young people and those of young people with other SENDs. In addition, there was no change in the mental health of parents as lockdown progressed and schools opened for face-to-face teaching. This is likely to be a result of different sources of mental health stressors. Earlier in the pandemic loss of support networks and specialist support affected parent mental health (Asbury et al., 2021) but later, once schools opened for face-to-face teaching, worries such as their child falling behind neurotypical peers and risk of infection from going to school were more prominent. These findings are interpreted in light of qualitative open comments from parents in Part 2 of this study (Asbury & Toseeb, 2021).

### Strengths and limitations

A number of strengths and limitations should be borne in mind when interpreting the findings of this study. To the best of the authors’ knowledge, it is the only study to investigate longitudinal trajectories of mental health in autistic young people, and their parents/carers, during the COVID-19 pandemic in the UK. Data were collected from the first day of the first UK lockdown and, where possible, families with followed up at multiple points to investigate the change mental health. A specific measure of anxiety, which was developed specifically for autistic populations, was used – thus providing confidence in measurement. One of the limitations is that missing data were dependent on educational placement and household income. Those receiving alternative provision and from low-income households were more likely to drop out compared to those from mainstream schools and high-income households. It may have been that families who were struggling the most may not have had the time to take part in multiple online surveys. In addition, parent/carer reports of young people’s mental health were used. Parents tend not to be fully aware of their child’s mental health needs as is evident from the low agreement between parent and child mental health measures. Self-report may also be problematic because autistic young people may not recognise problematic emotions and behaviours. Future research should adopt a multi-informant perspective to allow for a comprehensive account of mental health. A further limitation was that children who have few words were excluded from our sample because the measures were not appropriate for use with these children. Therefore, at best these findings only apply to verbal children and young people. There was also no comparison to pre-pandemic mental health. Finally, an opportunity sample was used. The findings presented here should be combined with evidence from other sources such as routinely collected administrative data and/or data from population cohort studies. This triangulating of evidence from multiple sources will help to address some of the drawbacks associated with online surveys making use of opportunity samples.

## Conclusions

In this large longitudinal study, we investigated how the mental health of autistic young people and their parents/carers changed during and after the first COVID-19 lockdown in the UK. As lockdown progressed and schools subsequently reopened for face-to-face teaching, symptoms of anxiety and depression remained stable for autistic young people. For those with other SENDs, symptoms of anxiety, but not depression, decreased during the same period. Parents’/carers’ psychological distress and well-being did not change as lockdown progressed. These findings suggest that the COVID-19 pandemic is likely to have disproportionately affected autistic young people. We attempt to explain and contextualise these findings in the second part of this study (Asbury & Toseeb, 2021).

## Supplemental Material

sj-docx-1-aut-10.1177_13623613221082715 – Supplemental material for A longitudinal study of the mental health of autistic children and adolescents and their parents during COVID-19: Part 1, quantitative findings Supplemental material, sj-docx-1-aut-10.1177_13623613221082715 for A longitudinal study of the mental health of autistic children and adolescents and their parents during COVID-19: Part 1, quantitative findings by Umar Toseeb and Kathryn Asbury in Autism

## References

[bibr1-13623613221082715] Alhuzimi T. (2021). Stress and emotional wellbeing of parents due to change in routine for children with Autism Spectrum Disorder (ASD) at home during COVID-19 pandemic in Saudi Arabia. Research in Developmental Disabilities, 108, Article 103822. 10.1016/j.ridd.2020.103822 33271447

[bibr2-13623613221082715] Althiabi Y. (2021). Attitude, anxiety and perceived mental health care needs among parents of children with Autism Spectrum Disorder (ASD) in Saudi Arabia during COVID-19 pandemic. Research in Developmental Disabilities, 111, Article 103873. 10.1016/j.ridd.2021.103873 33540358

[bibr3-13623613221082715] American Psychiatric Association. (2013). Diagnostic and statistical manual of mental disorders (5th ed.).

[bibr4-13623613221082715] Amorim R. Catarino S. Miragaia P. Ferreras C. Viana V. Guardiano M. (2020). The impact of COVID-19 on children with autism spectrum disorder. Revista de Neurologia, 71(8), 285–291. 10.33588/rn.7108.2020381 (Impacto de la COVID-19 en niños con trastorno del espectro autista.) 33034366

[bibr5-13623613221082715] Asbury K. Fox L. Deniz E. Code A. Toseeb U. (2021). How is COVID-19 affecting the mental health of children with special educational needs and disabilities and their families?. Journal of Autism and Developmental Disorders, 51(5), 1772–1780. 10.1007/s10803-020-04577-2 32737668 PMC7393330

[bibr6-13623613221082715] Asbury K. Toseeb U. (2022). A longitudinal study of the mental health of children and adolescents with autism and their parents during COVID-19: Part 2, qualitative findings. Autism, XX(X), XX–XX. 10.31234/osf.io/p2c5v PMC9805927 35669990

[bibr7-13623613221082715] Banerjee T. Khan A. Kesavan P. (2021). Impact of lockdown and school closure on children in special schools: A single-centre survey. BMJ Paediatrics Open, 5(1), Article e000981. 10.1136/bmjpo-2020-000981 PMC7888363 33665375

[bibr8-13623613221082715] Beesdo K. Knappe S. Pine D. S. (2009). Anxiety and anxiety disorders in children and adolescents: Developmental issues and implications for DSM-V. The Psychiatric Clinics of North America, 32(3), 483–524. 10.1016/j.psc.2009.06.002 19716988 PMC3018839

[bibr9-13623613221082715] Ben-Sasson A. Carter A. S. Briggs-Gowan M. J. (2009). Sensory over-responsivity in elementary school: Prevalence and social-emotional correlates. Journal of Abnormal Child Psychology, 37(5), 705–716. 10.1007/s10802-008-9295-8 19153827 PMC5972374

[bibr10-13623613221082715] Bronfenbrenner U. (1979). The ecology of human development: Experiments by nature and design. Harvard University Press.

[bibr11-13623613221082715] Chorpita B. F. Yim L. Moffitt C. Umemoto L. A. Francis S. E. (2000). Assessment of symptoms of DSM-IV anxiety and depression in children: A revised child anxiety and depression scale. Behaviour Research and Therapy, 38(8), 835–855. 10.1016/s0005-7967(99)00130-8 10937431

[bibr12-13623613221082715] Chou W. J. Wang P. W. Hsiao R. C. Hu H. F. Yen C. F. (2020). Role of school bullying involvement in depression, anxiety, suicidality, and low self-esteem among adolescents with high-functioning autism spectrum disorder. Frontiers in Psychiatry, 11, Article 9. 10.3389/fpsyt.2020.00009 PMC7005920 32082201

[bibr13-13623613221082715] Colizzi M. Sironi E. Antonini F. Ciceri M. L. Bovo C. Zoccante L. (2020). Psychosocial and behavioral impact of COVID-19 in autism spectrum disorder: An online parent survey. Brain Sciences, 10(6), Article 341. 10.3390/brainsci10060341 PMC7349059 32503172

[bibr14-13623613221082715] Davis N. O. Carter A. S. (2008). Parenting stress in mothers and fathers of toddlers with autism spectrum disorders: Associations with child characteristics. Journal of Autism and Developmental Disorders, 38(7), 1278–1291. 10.1007/s10803-007-0512-z 18240012

[bibr15-13623613221082715] DePape A. M. Lindsay S. (2014). Parents’ experiences of caring for a child with autism spectrum disorder. Qualitative Health Research, 25, 569–583. 10.1177/1049732314552455 25246329

[bibr16-13623613221082715] Goodman R. (1997). The strengths and difficulties questionnaire: A research note. Journal of Child Psychology and Psychiatry, 38(5), 581–586. 10.1111/j.1469-7610.1997.tb01545.x 9255702

[bibr17-13623613221082715] Green J. Gilchrist A. Burton D. Cox A. (2000). Social and psychiatric functioning in adolescents with Asperger syndrome compared with conduct disorder. Journal of Autism and Developmental Disorders, 30(4), 279–293. 10.1023/A:1005523232106 11039855

[bibr18-13623613221082715] Hoffman C. D. Sweeney D. P. Hodge D. Lopez-Wagner M. C. Looney L. (2009). Parenting stress and closeness: Mothers of typically developing children and mothers of children with autism. Focus on Autism and Other Developmental Disabilities, 24(3), 178–187. 10.1177/1088357609338715

[bibr19-13623613221082715] Hudson C. C. Hall L. Harkness K. L. (2019). Prevalence of depressive disorders in individuals with autism spectrum disorder: A meta-analysis. Journal of Abnormal Child Psychology, 47(1), 165–175. 10.1007/s10802-018-0402-1 29497980

[bibr20-13623613221082715] Ibrahim J. G. Molenberghs G. (2009). Missing data methods in longitudinal studies: A review. TEST, 18(1), 1–43. 10.1007/s11749-009-0138-x 21218187 PMC3016756

[bibr21-13623613221082715] Jenkinson R. Milne E. Thompson A. (2020). The relationship between intolerance of uncertainty and anxiety in autism: A systematic literature review and meta-analysis. Autism, 24(8), 1933–1944. 10.1177/1362361320932437 32564625 PMC7539603

[bibr22-13623613221082715] Kessler R. C. Barker P. R. Colpe L. J. Epstein J. F. Gfroerer J. C. Hiripi E. Howes M. J. Normand S. L. Manderscheid R. W. Walters E. E. Zaslavsky A. M. (2003). Screening for serious mental illness in the general population. Archives of General Psychiatry, 60(2), 184–189. 10.1001/archpsyc.60.2.184 12578436

[bibr23-13623613221082715] Lugo-Marín J. Gisbert-Gustemps L. Setien-Ramos I. Español-Martín G. Ibañez-Jimenez P. Forner-Puntonet M. Arteaga-Henríquez G. Soriano-Día A. Duque-Yemail J. D. Ramos-Quiroga J. A. (2021). COVID-19 pandemic effects in people with Autism Spectrum Disorder and their caregivers: Evaluation of social distancing and lockdown impact on mental health and general status. Research in Autism Spectrum Disorders, 83, Article 101757. 10.1016/j.rasd.2021.101757 PMC7904459 33649707

[bibr24-13623613221082715] Masi A. Mendoza Diaz A. Tully L. Azim S. I. Woolfenden S. Efron D. Eapen V. (2021). Impact of the COVID-19 pandemic on the well-being of children with neurodevelopmental disabilities and their parents. Journal of Paediatrics and Child Health, 57, 631–636. 10.1111/jpc.15285 33426739 PMC8014782

[bibr25-13623613221082715] Mazefsky C. A. Herrington J. Siegel M. Scarpa A. Maddox B. B. Scahill L. White S. W. (2013). The role of emotion regulation in autism spectrum disorder. Journal of American Academy of Child Adolescent Psychiatry, 52(7), 679–688. 10.1016/j.jaac.2013.05.006 PMC3719386 23800481

[bibr26-13623613221082715] Mutluer T. Doenyas C. Aslan Genc H. (2020). Behavioral implications of the Covid-19 process for autism spectrum disorder, and individuals’ comprehension of and reactions to the pandemic conditions. Frontiers in Psychiatry, 11, Article 561882. 10.3389/fpsyt.2020.561882 PMC7701051 33304279

[bibr27-13623613221082715] Nonweiler J. Rattray F. Baulcomb J. Happé F. Absoud M. (2020). Prevalence and associated factors of emotional and behavioural difficulties during COVID-19 pandemic in children with neurodevelopmental disorders. Children, 7(9), Article 128. 10.3390/children7090128 PMC7552706 32899799

[bibr28-13623613221082715] Nooraee N. Molenberghs G. Ormel J. Van den Heuvel E. R. (2018). Strategies for handling missing data in longitudinal studies with questionnaires. Journal of Statistical Computation and Simulation, 88(17), 3415–3436. 10.1080/00949655.2018.1520854

[bibr29-13623613221082715] Nuñez A. Le Roy C. Coelho-Medeiros M. E. López-Espejo M. (2021). Factors affecting the behavior of children with ASD during the first outbreak of the COVID-19 pandemic. Neurological Sciences: Official Journal of the Italian Neurological Society and the Italian Society of Clinical Neurophysiology, 42(5), 1675–1678. 10.1007/s10072-021-05147-9 PMC7914113 33641028

[bibr30-13623613221082715] O’Sullivan K. Clark S. McGrane A. Rock N. Burke L. Boyle N. Joksimovic N. Marshall K. (2021). A qualitative study of child and adolescent mental health during the COVID-19 pandemic in Ireland. International Journal of Environmental Research and Public Health, 18(3), Article 1062. 10.3390/ijerph18031062 PMC7908364 33504101

[bibr31-13623613221082715] Pecor K. W. Barbayannis G. Yang M. Johnson J. Materasso S. Borda M. Garcia D. Garla V. Ming X. (2021). Quality of life changes during the COVID-19 pandemic for caregivers of children with ADHD and/or ASD. International Journal of Environmental Research and Public Health, 18(7), Article 3667. https://www.mdpi.com/1660-4601/18/7/3667 10.3390/ijerph18073667 PMC8037979 33915884

[bibr32-13623613221082715] Pisula E. (2007). A comparative study of stress profiles in mothers of children with autism and those of children with Down’s syndrome. Journal of Applied Research in Intellectual Disabilities, 20(3), 274–278. 10.1111/j.1468-3148.2006.00342.x

[bibr33-13623613221082715] Quintero N. McIntyre L. L. (2010). Sibling adjustment and maternal well-being: An examination of families with and without a child with an autism spectrum disorder. Focus on Autism and Other Developmental Disabilities, 25(1), 37–46. 10.1177/1088357609350367 21037802 PMC2966315

[bibr34-13623613221082715] Raw J. A. L. Waite P. Pearcey S. Shum A. Patalay P. Creswell C. (2021). Examining changes in parent-reported child and adolescent mental health throughout the UK’s first COVID-19 national lockdown. Journal of Child Psychology and Psychiatry, 62, 1391–1401. 10.1111/jcpp.13490 34327726 PMC8447308

[bibr35-13623613221082715] Rodgers J. Wigham S. McConachie H. Freeston M. Honey E. Parr J. R. (2016). Development of the anxiety scale for children with autism spectrum disorder (ASC-ASD). Autism Research, 9(11), 1205–1215. 10.1002/aur.1603 26887910

[bibr36-13623613221082715] Sedgewick F. Leppanen J. Tchanturia K. (2019). The Friendship Questionnaire, autism, and gender differences: A study revisited. Molecular Autism, 10(1), Article 40. 10.1186/s13229-019-0295-z PMC6883660 31798817

[bibr37-13623613221082715] Siracusano M. Segatori E. Riccioni A. Emberti Gialloreti L. Curatolo P. Mazzone L. (2021). The impact of COVID-19 on the adaptive functioning, behavioral problems, and repetitive behaviors of Italian children with autism spectrum disorder: An observational study. Children, 8(2), Article 96. https://www.mdpi.com/2227-9067/8/2/96 10.3390/children8020096 PMC7913091 33540683

[bibr38-13623613221082715] StataCorp. (2021). Stata statistical software: Release 17.

[bibr39-13623613221082715] Tennant R. Hiller L. Fishwick R. Platt S. Joseph S. Weich S. Parkinson J. Secker J. Stewart-Brown S. (2007). The Warwick-Edinburgh Mental Well-being Scale (WEMWBS): Development and UK validation. Health and Quality of Life Outcomes, 5, Article 63. 10.1186/1477-7525-5-63 PMC2222612 18042300

[bibr40-13623613221082715] Toseeb U. (2021). Sibling conflict during COVID-19 in families with special educational needs and disabilities. British Journal of Educational Psychology, 92, 319–339. 10.1111/bjep.12451 34423422 PMC8646725

[bibr41-13623613221082715] Toseeb U. Asbury K. Code A. Fox L. Deniz E. (2020). Supporting families with children with special educational needs and disabilities during COVID-19. PsyArXiv Preprints. 10.31234/osf.io/tm69k PMC7393330 32737668

[bibr42-13623613221082715] Toseeb U. McChesney G. Oldfield J. Wolke D. (2020). Sibling bullying in middle childhood is associated with psychosocial difficulties in early adolescence: The case of individuals with autism spectrum disorder. Journal of Autism and Developmental Disorders, 50, 1457–1469. 10.1007/s10803-019-04116-8 31332675 PMC7211196

[bibr43-13623613221082715] Toseeb U. McChesney G. Wolke D. (2018). The prevalence and psychopathological correlates of sibling bullying in children with and without autism spectrum disorder. Journal of Autism and Developmental Disorders, 48, 2308–2318. 10.1007/s10803-018-3484-2 29423609 PMC5996014

[bibr44-13623613221082715] van Steensel F. J. A. Bögels S. M. de Bruin E. I . (2013). Psychiatric comorbidity in children with autism spectrum disorders: A comparison with children with ADHD. Journal of Child and Family Studies, 22(3), 368–376. 10.1007/s10826-012-9587-z 23524401 PMC3602612

[bibr45-13623613221082715] van Steensel F. J. A. Bogels S. M. Perrin S . (2011). Anxiety disorders in children and adolescents with autistic spectrum disorders: A meta-analysis. Clinical Child and Family Psychology Review, 14(3), 302–317. 10.1007/s10567-011-0097-0 21735077 PMC3162631

[bibr46-13623613221082715] Vasa R. A. Singh V. Holingue C. Kalb L. G. Jang Y. Keefer A. (2021). Psychiatric problems during the COVID-19 pandemic in children with autism spectrum disorder. Autism Research, 14, 2113–2119. 10.1002/aur.2574 34231323 PMC8420610

[bibr47-13623613221082715] Wang L. Li D. Pan S. Zhai J. Xia W. Sun C. Zou M. (2021). The relationship between 2019-nCoV and psychological distress among parents of children with autism spectrum disorder. Globalization and Health, 17(1), Article 23. 10.1186/s12992-021-00674-8 PMC7905970 33632259

