# Supplementary material for: A longitudinal study of the mental health of autistic children and adolescents and their parents during COVID-19: Part 1, quantitative findings
Source: Autism. 2022 Jun 6;27(1):105–16. doi: 10.1177/13623613221082715 (PMC9805925; doi:10.1177/13623613221082715)
Supplement: sj-docx-1-aut-10.1177_13623613221082715 – Supplemental material for A longitudinal study of the mental health of autistic children and adolescents and their parents during COVID-19: Part 1, quantitative findings [file sj-docx-1-aut-10.1177_13623613221082715.docx]

**Supplementary Materials**

**Missing Data**

Sample attrition was calculated as the proportion of participants who took part in all previous time points. The sample attrition was as follows: T2 - 56% (173 parents took part at T1 but not T2), T3 – 72% (288 parents took part at T1 or T2 but not T3), and T4 – 63% (326 parents took part at T1, T2, or T3 but not T4). There were 260 (50%) participants who only took part at one-time point, 158 (31%) who took part at two-time points, 53 (10%) who took part at three-time points, and 46 (9%) who took part at all four-time points.

There were missing data at each time point (Time 1 40%, Time 2 56%, Time 3 63%, Time 4 63%). These high levels of missing data were reflective of the study design and, to some extent, were expected. The study design meant that there were two sources of missing data: 1) participants who took part in earlier but not later time points (i.e. sample attrition) and 2) participants who took part in later but not earlier time points (i.e. those who joined the study at the later waves of data collection). A series of chi-squared tests were run to test whether the number of time points parent carers completed the questionnaire was associated with variables of interest. This was used as a proxy to determine whether missingness was dependent on key variables. Missingness was not different based on whether the young person had autism (χ2(509)=.16, p=.983), young person’s sex (χ2(504)=1.24, p=.744), young person’s ethnicity (χ2(508)=2.11, p=.551), or whether they had an education, health, and care plan (χ2(509)=1.84, p=.607). Missingness was different based on school type (χ2(509)=8.50, p=.037) and household income (χ2(500)=9.84, p=.020). Parents of children who attended a non-mainstream school, on average, took part at more time points compared to those in mainstream schools. Those from low-income households took part at fewer time points compared to those not from low-income households. The maximum likelihood estimator was used in all statistical models to deal with missing data.

**Table S1.**

*Type of Special Educational Needs and Disabilities (SEND) as Reported by the Parent Caregiver*

|  | **Overall N (%)** | **Time 1 N (%)** | **Time 2 N (%)** | **Time 3 N (%)** | **Time 4 N (%)** |
| --- | --- | --- | --- | --- | --- |
| **Type of SEND** |  |  |  |  |  |
| Autism Spectrum Disorder | 389 (75%) | 250 (81%) | 184 (80%) | 130 (69%) | 144 (75%) |
| Social, Emotional, and Mental Health Difficulties | 177 (34%) | 115 (37%) | 90 (39%) | 56 (30%) | 64 (34%) |
| Attention Deficit Hyperactivity Disorder | 116 (22%) | 74 (24%) | 48 (21%) | 40 (21%) | 46 (24%) |
| Dyslexia | 75 (15%) | 41 (13%) | 34 (15%) | 26 (14%) | 22 (12%) |
| Developmental Coordination Disorder | 56 (11%) | 32 (10%) | 28 (12%) | 25 (13%) | 28 (15%) |
| Developmental Language Disorder | 46 (9%) | 37 (12%) | 22 (10%) | 21 (11%) | 20 (10%) |
| Speech Disorder or Impediment | 44 (9%) | 27 (9%) | 17 (7%) | 14 (7%) | 18 (9%) |
| Physical Disability | 34 (7%) | 22 (7%) | 13 (6%) | 13 (7%) | 7 (4%) |
| Attention Deficit Disorder | 37 (7%) | 25 (8%) | 14 (6%) | 17 (9%) | 12 (6%) |
| Sensory Processing Disorder | 33 (6%) | 12 (4%) | 9 (4%) | 18 (10%) | 18 (9%) |
| Other^+^ | 91 (18%) | 47 (15%) | 51 (22%) | 28 (15%) | 31 (16%) |
| **Co-Occurrence of SENDs** |  |  |  |  |  |
| One SEND | 215 (42%) | 118 (38%) | 84 (37%) | 89 (47%) | 78 (41%) |
| Two SENDs | 128 (25%) | 77 (25%) | 58 (25%) | 39 (21%) | 50 (26%) |
| Three SENDs | 102 (20%) | 66 (21%) | 54 (24%) | 31 (16%) | 36 (19%) |
| Four or more SENDs | 72 (14%) | 49 (16%) | 33 (14%) | 30 (16%) | 27 (14%) |

**Note.** Parents were asked to select all that applied to their child from a list. ^+^Table only includes types of special educational needs and disabilities that were endorsed by >5% of parents (overall across all time points). The remainder were included in the other category, which includes conduct disorder, dyscalculia, Down syndrome, epilepsy, hearing impairment, moderate learning difficulties, global developmental delay, and visual impairment.

**Table S2**

*Regression Model for Anxiety Symptoms– Split by Group*

|  | **Autism** | **SEND** |
| --- | --- | --- |
| Wald χ^2^ | χ^2^(7, 367)=35.36, p<.001 | χ^2^(7, 114)=13.69, p=.057 |
| **Predictor** |  |  |
| Linear Time | -.06 [-.30, .19] | **-.76 [-1.26, -.26]^**^** |
| Age | **.77 [.38, 1.15]^***^** | .15 [-.48, .78] |
| Boy | **4.70 [-7.70, -1.70]^**^** | -.38 [-.52, 4.47] |
| Ethnic Minority | -2.14 [-6.95, 2.66] | -.6.30 [-12.51, -.08]^*^ |
| Low-income | 2.57 [-.08, 5.21] | 1.68 [-2.83, 6.20] |
| EHCP | -.63 [-3.74, 2.48] | -.49 [-5.42, 4.43] |
| Non-mainstream school | -3.07 [-6.08, -.06]^*^ | .28 [-4.96, 5.51] |

**Note.** ^*^p<.05, ^**^p<.01, ^***^p<.001. Values are unstandardised beta coefficients [95% confidence intervals]. Predictors that remained significant after Bonferroni correction are in bold.

**Table S3**

*Regression Model for Anxiety Symptoms – Split by Time*

|  | **Time 1** | **Time 2** | **Time 3** | **Time 4** |
| --- | --- | --- | --- | --- |
| Wald χ^2^ | χ^2^(7, 285)=46.23, p<.001 | χ^2^(7, 207)=24.74, p<.001 | χ^2^(7, 167)=47.66, p<.001 | χ^2^(7, 180)=49.89, p<.001 |
| **Predictor** |  |  |  |  |
| Autism | **8.98 [4.91, 13.05]^***^** | **8.48 [3.78, 13.18]^***^** | **11.97 [7.76, 16.19]^***^** | **15.78 [11.05, 20.51]^***^** |
| Age | **.73 [.22, 1.24]^**^** | .48 [-.09, 1.06] | .39 [-.20, .98] | .31 [-.31, .94] |
| Boy | -3.18 [-6.68, .32] | -3.08 [-7.25, 1.08] | -3.90 [-7.88, .08] | -4.47 [-9.06, .12] |
| Ethnic Minority | -6.01 [-11.69, -.34]^*^ | -6.33 [-12.62, -.03] | -3.87 [-10.12, 2.38] | -2.84 [-9.46, 3.77] |
| Low-income | 1.80 [-1.39, 4.99] | 1.57 [-2.06, 5.20] | 4.29 [.49, 8.09]^*^ | 2.99 [-1.10, 7.09] |
| EHCP | -2.12 [-6.00, 1.76] | -.72 [-4.95, 3.51] | -3.85 [-8.13, .42] | -1.53 [-5.98, 2.91] |
| Non-mainstream school | -3.39 [-7.06, .28] | -2.12 [-6.34, 2.10] | 1.12 [-3.20, 5.43] | -1.72 [-6.27, 2.82] |

**Note.** ^*^p<.05, ^**^p<.01, ^***^p<.001. Values are unstandardised beta coefficients [95% confidence intervals]. Predictors that remained significant after Bonferroni correction are in bold.
